# Rating of perceived exertion in continuous sports: a scoping review with evidence gap map

**DOI:** 10.3389/fspor.2025.1553998

**Published:** 2025-07-15

**Authors:** Gonçalo Torres, Filipe Maia, Fábio Yuzo Nakamura, Henrique Pereira Neiva, Ana Sousa

**Affiliations:** ^1^ Research Center in Sports Sciences, Health Sciences and Human Development, CIDESD, Vila Real, Portugal; ^2^ Department of Sport Sciences, University of Maia, Maia, Portugal; ^3^ Department of Sport Sciences, University of Beira Interior, Covilhã, Portugal

**Keywords:** competition, physiology, cyclic sports, effort, internal load

## Abstract

**Introduction:**

Rating of perceived exertion (RPE) is widely used for assessing training load in sports due to its validity, simplicity, and utility. Despite its broad application, the diverse contexts and methodologies in which it is used warrant a comprehensive review of the existing evidence.

**Objective:**

This scoping review aims to map the current evidence on the use of RPE, focusing on its application, measurement methods, and reliability across different continuous sports.

**Methods:**

Databases PubMed, SportDiscus (via EBSCO), Scopus, and Web of Science (core collection) were systematically searched until 22 May 2025 using the search terms: ([(RPE) OR (rating of perceived exertion) OR (Borg Scale)] AND (load) AND [(sports) OR (exercise) OR athletes]) Studies were included in this review if they complied with the following criteria: (1) conducted in continuous modes of exercise, (2) considering the comparison with other internal and external load measures, (3) when healthy and trained athletes were studied, (4) written in English language.

**Results:**

A total of 234 studies involving 4,388 athletes were included in this review. Findings indicated that RPE is primarily used in training control and prescription (∼35%). A small number of studies focused directly on female athletes (∼7%), similarly master (∼1%) and elite athletes (∼13%) research was scarce.

**Conclusion:**

The findings suggest that although RPE is a valuable tool, variability in application across different exercise settings highlights the necessity to standardize its guidelines. Future research should focus on assessing the use of RPE in under-represented continuous sports.

**Systematic Review Registration:**

https://doi.org/10.17605/OSF.IO/C9PW6.

## 1 Introduction

The rating of perceived exertion (RPE) scale has revolutionized the concept of training load monitoring in sports, due to its validity, practicality, and cost-effectiveness (1). The concept of RPE, first introduced by Borg (2) in the late 1950s, has gained increasing attention in sport and exercise science literature, evolving into a widely used tool to assess onés conscious sensation of how hard, heavy and strenuous a physical work is. Despite its importance and usefulness in monitoring and prescribing exercise intensity, its neurophysiological bases are poorly understood. The mechanisms underlying generation of RPE, extend beyond the direct feedback from the cardiovascular, respiratory and musculoskeletal systems as was popularly proposed by Proske (3) and Dempsey (4). Recent research suggests that RPE is more likely driven by brain processing of neural signals -corollary discharges- generated by premotor/motor areas of the cerebral cortex to sensory areas when they generate central motor commands to initiate and sustain voluntary skeletal muscle contractions (5), rather than purely physiological feedback from the working systems. This model confirms the definition of exertion (6) as “the degree of heaviness and strain experienced in physical work”. Such explanation would corroborate with the verbal descriptors chosen by Borg for their RPE scales as he used “heavy/hard” as opposed to “pleasant/unpleasant” or “comfortable/uncomfortable” as ratings of hedonicity (7).

To use the initial version of this tool, individuals were instructed to rate their perceived exertion during or after a task on a 6–20 scale, which was designed to range between the resting heart rate (HR) of 60 beats/min to its theoretical maximal of 200 beats/min for a 20-year-old person (e.g., a rating of 13 would correspond to approximately an HR of approximately 130 beats/min) (8). Subsequently, Borg introduced a new category scale with ratio properties, the CR-10 Scale, which ranges from 0 to 10. In this version, the numbers are anchored to verbal expressions, and a true zero point was established to facilitate understanding (9).

RPE is a subjective measure influenced by both physiological and psychological factors. While it correlates with objective markers such as HR and blood lactate concentration (Bla) (10), it primarily reflects an individual's subjective perception of the heaviness of a given workload, being influenced by fatigue and mental states during exercise (1). Psychological factors, such as motivation and mood, can also impact on how effort is perceived, with mental fatigue and task engagement often influencing RPE) (11). This subjective nature of RPE allows it to capture an athlete's internal experience of exertion, providing a more comprehensive understanding of training load than physiological markers alone (12). As a result, RPE is regarded as a valuable tool for optimizing training and monitoring athletes' load.

RPE, especially in its Session-RPE (S-RPE) application (1), has become a cornerstone for monitoring and managing training loads (TL). By multiplying RPE scores by the session duration (in minutes), coaches and practitioners can derive metrics such as Training Monotony and Strain, enabling them to optimize periodization and mitigate the risks of undertraining, non-functional overreaching, and overtraining (13). These conditions have been linked to negative outcomes, including increased illness risk, poor performance, and overuse injuries (14). High training loads without adequate recovery may lead to maladaptation and loss of performance. Conversely, insufficient load may fail to stimulate the body to adapt and improve physical performance (15). Therefore, the importance of training load monitoring in various sports has been the focus of numerous studies (16), emphasizing the need for sports coaches to manage and adjust training programs with precision.

In this regard, comparing the agreement between the S-RPE planned by coaches and the S-RPE reported by athletes has been the subject of interest and investigation. A systematic review with meta-analysis (17). showed good agreement between coaches and athletes regarding overall, moderate, and hard RPE, as well as S-RPE values. However, it revealed a discrepancy in lower RPE and S-RPE values, indicating that coaches perceived their planned “easy” sessions as easier than athletes did.

While RPE has been widely applied, research appears to have predominantly focused on male professional athletes in team sports, particularly soccer (18), and on continuous modalities such as cycling, running, and swimming (16). Collectively, this narrow scope limits our understanding of RPE's broader applications and interactions, particularly: (i) its use across different exercise intensity domains, (ii) its role in longitudinal study settings, and (iii) its application to female athletes (19) and elite athletes (20), especially those participating in underrepresented continuous sports (15). These latter are characterized by uninterrupted physical activity with minimal or no pauses during performance, involving sustained effort over their respective duration (16). Given the current gaps in literature, a scoping review, combined with an evidence gap map (EGM), is highly relevant for evaluating the existing body of knowledge on RPE, identifying prevailing practices, and highlighting areas for future research. Scoping reviews are particularly useful for mapping key concepts, summarizing evidence from diverse study designs, and identifying knowledge gaps in emerging or complex fields. By offering a comprehensive overview, they can guide future research priorities and inform practical applications.

This scoping review aims to explore the various applications of RPE across different continuous sports, settings, and contexts in athlete populations while providing insights into its use, limitations, and potential for enhancing training prescription and monitoring.

## 2 Methods

This scoping review was conducted in accordance with the Preferred Reporting Items for Systematic Reviews and Meta-Analyses (PRISMA) extension for scoping reviews (PRISMA-ScR) (21). Additionally, the study protocol was developed and pre-registered by the authors on the Open Science Framework (osf.io/k86eb).

### 2.1 Eligibility criteria

The inclusion criteria were determined based on published peer-reviewed articles, with no restrictions on the year of publication but limited to the English language due to the specialized skills, time, and funding required for accurate translation and interpretation of non-English studies. Similarly, grey literature was excluded to ensure methodological rigor and consistency, as non-peer-reviewed sources often lack detailed reporting and may introduce bias.

These criteria were structured according to the Participants, Intervention, Comparators, Outcomes and Study Design (PICOS) framework:

(P) Healthy athletes of any age, sex, engaged in continuous modes of exercise and classified as tier 2 (trained/developmental), or superior, according to the definition proposed by McKay et al. Being RPE a tool that requires familiarity with itself, as well as with the exercise in question, subjects classified as tier 0 or 1 (sedentary or recreationally active) were excluded from this analysis as they may not have the desired experience to accurately assess their exertion (22) and therefore, conclusions derived from these populations should not be extrapolated to trained individuals). Studies involving injured (e.g., returning to practice) or disabled athletes (e.g., paralympic swimming) were excluded as well. Since the goal was to provide an overview of the research field, no minimum number of participants per study was required.
(I) Acute (single session or multiple sessions with less than 1 week of interval between each other) or Chronic (multiple sessions with 1 week, or longer, of interval between each other) interventions that would use RPE as internal load measure during or following physical activities.
(C) Comparators were not compulsory (because we were not directly comparing effectiveness or efficacy of different protocols), although if available, control groups were used in comparison to conditions such as hypoxic training or heat acclimatation.
(O) Effectiveness of RPE in monitoring training intensity, relationship between RPE and performance metrics, or psychological measures.
(S) Observational and experimental designs, including randomized controlled trials (crossover, parallel-group, pre-post, single group non-randomized studies) and case studies.

### 2.2 Search strategy, information sources and selection process

The databases PubMed, SportDiscus (via EBSCO), Scopus, and Web of Science (core collection) were consulted on September 20th, 2024, and updated on May 22nd, 2025, using the search terms: [(RPE) OR (rating of perceived exertion) OR (Borg Scale)] AND (load) AND [(sports) OR (exercise) OR (athletes)].

The results of the search were exported to the reference manager software EndNote 20™ (Clarivate Analytics, Philadelphia, PA), and duplicate records were automatically removed and manually confirmed. Two authors (GT, FM) independently screened all the retrieved articles during two screening rounds: first by title and abstract reading, and second by full-text analysis. A third investigator (AS) served as an arbitrator to solve discrepancies.

### 2.3 Data extraction

Two authors (GT, FM) independently extracted the data from the included studies into a Microsoft Excel document (Microsoft Corp.) that summarized the following items: (i) authors, (ii) year, (iii) number of participants and characteristics (e.g., sex, body mass, age, height), (iv) competitive tier of participants (from 2 to 5), (vi) study protocol, (v) study main outcomes, (vi) sport and its specialty (if applicable), (vii) main results, (viii) study design (e.g., observational, randomized crossover), and (ix) study category (e.g., physiological, psychological, performance outcomes, training monitoring). A third author (AS) was consulted to resolve discrepancies.

### 2.4 Data synthesis method

A narrative synthesis of the results was performed according to the previously described data items. To provide an overview of the existing scientific evidence on the topic and current research gaps, the evidence gap mapping was conducted. This mapping visually represents the distribution of evidence, allowing for an intuitive understanding of both the current body of research and areas requiring further investigation. In the EGM, total area of the circle indicates the number of studies corresponding to each category, providing a clear depiction of trends and disparities across the included studies.

## 3 Results

### 3.1 Study selection

**Figure 1:**
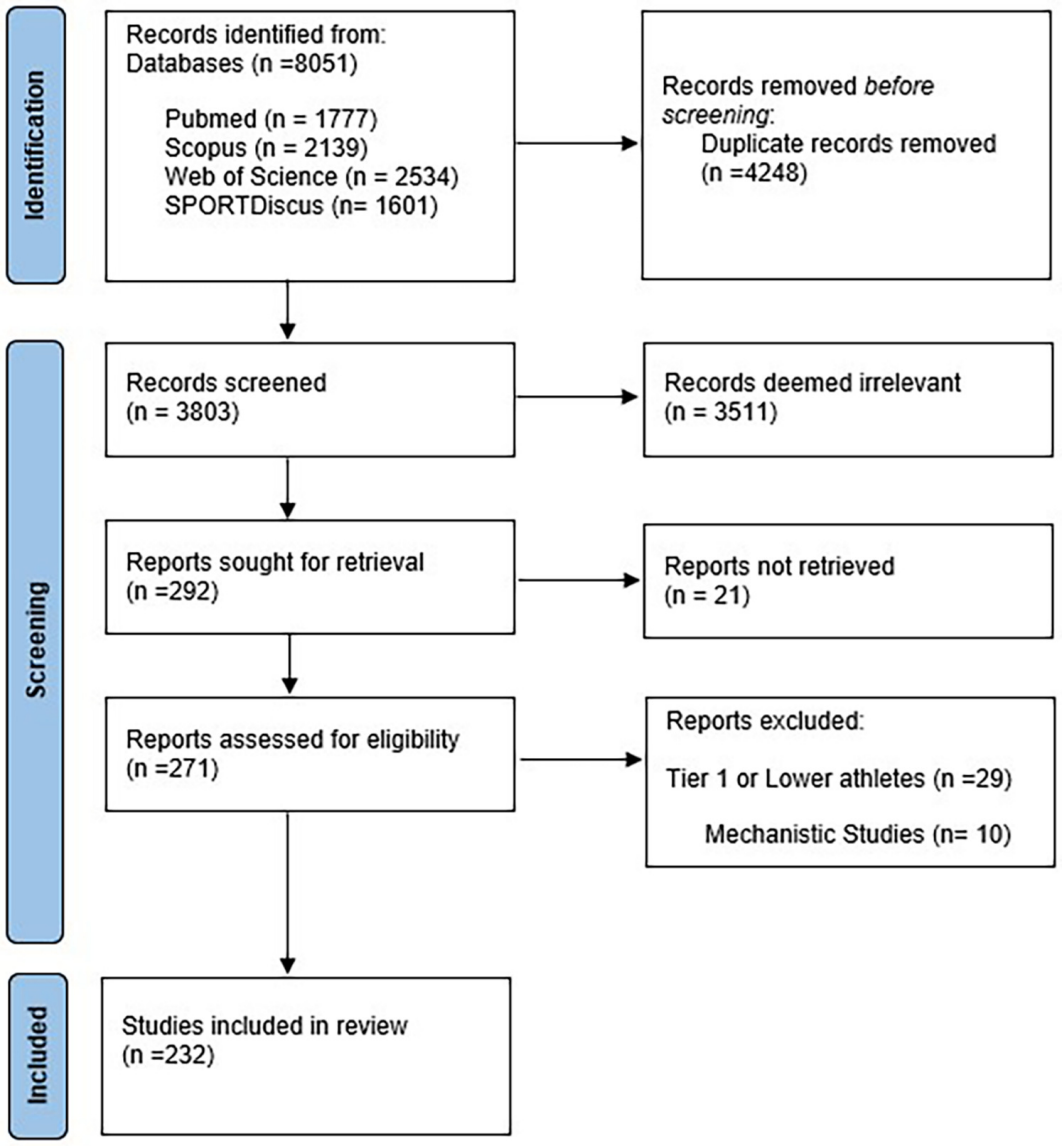
PRISMA flow diagram used for the article search.

The Prisma flow diagram (Figure 1) illustrates the process of study selection for this scoping review. An initial search of the databases identified 8,051 articles, which were exported to reference manager software EndNote 20™ (Clarivate Analytics, Philadelphia, PA). After removing duplicate records, 3,803 articles remained for screening. During the first screening round (title and abstract), 3,511 articles were excluded due to irrelevant topics or incorrect study design, leaving 292 articles for full-text review. In the second screening round, 58 articles were excluded because they did not meet the inclusion criteria related to participants or study design. In the end, 234 articles met the eligibility criteria (representing 4,388 participants) and were included in this scoping review, as shown in the flow diagram in Figure 1.

### 3.2 Characteristics of included studies

#### 3.2.1 Publication year and study designs

The 234 studies (23–254) included in this review were published between 1985 and 2025, indicating a growing trend in research on this topic over the years. The early exploration of this topic was limited, with only 10 studies published between 1985 and 2000. From 2001 onward, the number of research publications progressively increased, with 10 studies published between 2001 and 2005 and 16 studies between 2006 and 2010. This growth became more pronounced over the last decade and a half. Between 2011 and 2015, there was a significant increase in research on this topic, with 46 studies published. The trend peaked in the 2016–2020 period, during which 81 studies were conducted, making it the most productive 5-year interval to date. The subsequent 5-year period (2021–2025) showed a slight decrease in the research topic, with 71 studies being identified. Although it still represents a high level of research activity, maintaining a stable interest in the field, it may also reflect the stabilization of the research topic.

Most studies employed an experimental design (156 studies), while 78 presented an observational nature. Within experimental studies, different methodological approaches have been used. The largest subset (72 studies) used a single-group design, focusing on within-group changes without the use of a comparison group, or did not randomize subjects. The randomized-crossover design was employed in 57 studies, comparing different interventions within the same participants in a random and counterbalanced order. Finally, 27 studies made use of independent and randomized group designs, comparing outcomes across distinct participant groups to assess the effects of several distinct interventions.

### 3.3 Funding and competing interests

Among the 234 studies in this review, 80 (∼34%) declared the receipt of funding for conducting research, whereas 44 (∼19%) studies reported no funding. However, a total of 110 (∼47%) studies did not provide any information regarding funding sources, indicating a need for improved transparency in reporting financial support.

In the context of competing interests, 111 (∼48%) studies explicitly stated that there were no conflicts of interest to disclose. Conversely, 122 studies (∼52%) did not report any information on competing interests, leaving this important aspect of transparency unaddressed in over half of the included studies.

### 3.4 Sample size, age and sex

One hundred and nine studies included between 10 and 19 participants, while 54 studies involved 5–9 participants, and 41 studies had sample sizes ranging from 20 to 29. A smaller number of studies had larger sample sizes, with only 18 studies involving over 40 participants, and 5 studies involving between 30 and 39 participants. In contrast, 7 studies had very small sample sizes (case studies), with 1–4 participants.

Sex distribution among study participants was unevenly distributed. One hundred and nine research articles included only male participants, reflecting a notable male dominance in the topic. In 88 studies, both male and female participants were included, in 17 studies only female participants were included, and in 20 studies, the sex of participants was not described.

The age distribution of participants shows a predominance of younger populations. Sixty-eight studies involved the participation of adults (24–30 years old), highlighting an interest in this particular age range within this topic. Early adulthood (18–23 years old), is also well represented with 63 studies. Adolescent participants (12–17 years old) were included in 36 studies, and middle-aged adults (31–44 years) were included in 50 studies. Moreover, three studies explored the use of RPE in master's athletes (45+). In 14 studies, participant ages were not specified.

### 3.5 Sports, competitive level and type of intervention

The most frequently studied sports were cycling (83 studies) and running (74 studies). Other studied sports included swimming (34 studies), rowing (15 studies), triathlon (8 studies), and a smaller representation in skiing (7 studies), kayaking (5 studies), and canoe slalom (2 studies). Finally, 6 studies included participants from more than one sport (classified as multi-sport). Table 1 shows the distribution of RPE application method, key outcomes, measures and average sample sizes.

**Table 1:** Representation of RPE application method, key outcome, measures and average sample size by sport.

Sport	RPE application method	Key outcome	Measures	Average sample sizes
Running	RPE estimation	Physiological	RPE, HR, Bla, VO2	17
Cycling	RPE estimation	Performance	RPE, HR, Bla, power output	23
Swimming	RPE estimation	Training monitoring	RPE, HR, Bla, time trial	19
Rowing	Session RPE method	Training monitoring	RPE, HR, Bla	17
Triathlon	RPE estimation	Physiological	RPE, HR, Bla	15
Skiing	RPE estimation	Performance	RPE, HR, Bla	12
Kayak	RPE estimation	Physiological	RPE, HR, BLa	9
Canoe slalom	Session RPE method	Training monitoring	RPE, HRV	21

The studies included athletes across a wide range of competitive levels based on McKay et al. tier classification. Specifically: tier 2 (trained/developmental) athletes were included in 97 studies; tier 3 (highly trained/national level) athletes were the second most represented (74 studies); tier 4 (elite/international level) athletes were included in 31 studies; and 2 studies were conducted with tier 5 athletes. Additionally, some studies included participants in more than one competitive tier, with 11 studies grouping athletes from both tier 2 and 3, and 15 studies grouping both tier 3 and 4 athletes.

Studies employing acute (up to 1 week: 121) and chronic (longer than 1 week: 113) interventions were evenly distributed.

### 3.6 Outcomes of the included studies

#### 3.6.1 Overview of outcomes

Several outcomes were assessed in this study, comprising different aspects of sports performance. Specifically, studies were grouped into 5 major domains: physiological [including, but not limited to, parameters such as HR, maximal oxygen uptake (VO_2max_), and lactate threshold—78] training monitoring (focusing on aspects such as optimizing training intensity and volume—86), performance (measuring improvements in competitive outcomes such as time to exhaustion or power output—58), psychological (addressing mental aspects of sports—7), and biomechanical (focusing on movement patterns and their relationship with perceived exertion—5). This distribution reflects a holistic approach, with a strong emphasis on physiological and training-related outcomes.

### 3.7 Outcomes assessed

Among the most observed physiological outcomes, HR and Bla were present in, respectively, 126 and 84 studies. Additionally, performance measurements were also highly prevalent, being VO_2max_ present in 53 studies, power output in 52 studies, time trial in 35 studies and jumping performance (countermovement jump) in 7 studies. Furthermore, RPE was collected and compared consistently with the training-impulse (TRIMP) method (28 studies), and, in fewer studies, with the profile of mood states (POMS) scale (12 studies). In a lesser extent, sleeping parameters were monitored in 7 studies.

**Figure 2:**
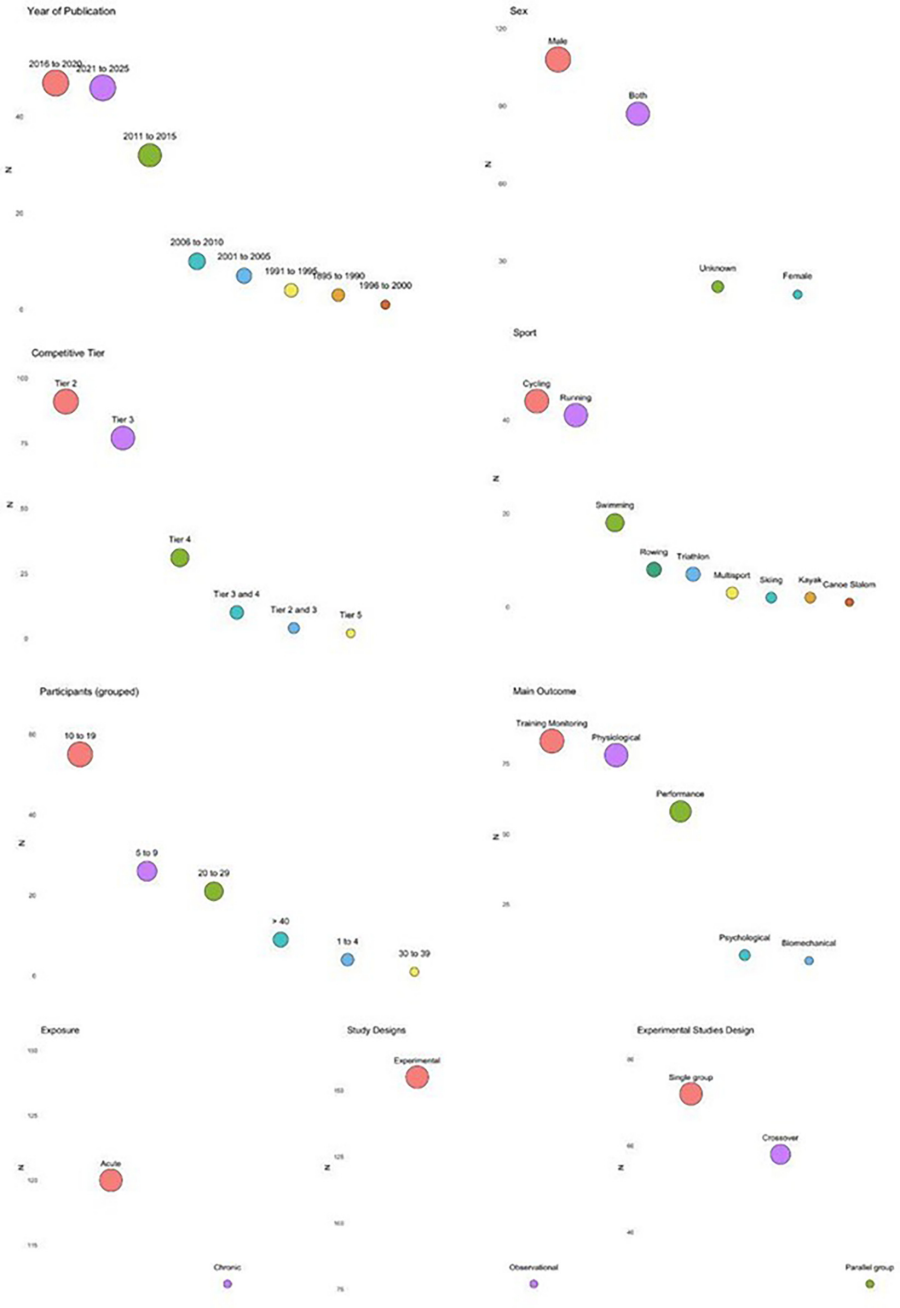
EGM of RPE research in continuous modes of exercise in healthy athletes. Rows represent primary outcome measures. Circle size within each cell reflects the relative magnitude of evidence for a given outcome compared to others, with larger circles indicating greater study volume and smaller circles fewer studies.

Figure 2 shows the EGM synthesizing the patterns and gaps previously described.

Examination of the EGM reveals several important findings: (1) there was a predominance of studies with small sample sizes (up to 20 participants), while larger scale trials (>40) were scarce; (2) the majority of research involved male participants, whilst studies focusing solely on females are lacking; (3) Running and Cycling were the most commonly studied sports, whereas Kayak and Canoe Slalom were clearly underrepresented; (4) most studies focused on tier 2 and 3 athletes, with limited research involving tier 5 athletes; (5) the majority of studies were acute in nature, typically lasting less than 1 week.

## 4 Discussion

RPE is implemented extensively in multiple sports and different contextual settings. Notwithstanding the high number of studies investigating this tool in different modes of exercise and populations, using many protocols and methodologies, an updated and reliable summary becomes paramount. To shape future research and funding strategies, we conducted a comprehensive review of the current literature to map existing studies and highlight trends and gaps in knowledge regarding RPE in healthy athletes, of both genders, from continuous sports, with a minimum competitive level of tier 2 or higher (255). Such summary is essential for informing future research directions and funding strategies, ensuring that RPE remains a reliable tool in monitoring training intensity.

### 4.1 Subjects

A persistent limitation in sports science research is the prevalence of small sample sizes, with many studies involving 50 or fewer athletes (256, 257). While this may be partly attributed to logistical constraints, particularly the recruitment of tier 4 and 5 athletes who face intensive training schedules and limited availability (255) its methodological implications are substantial. Small samples diminish statistical power, increasing the risk of Type II errors and reducing the likelihood that significant findings can be replicated (258). This contributes to the broader reproducibility concerns in sports science and raises questions about the external validity of current evidence.

For practitioners, this creates a gap between research and application: coaches and athletes are often forced to rely on evidence derived from suboptimal study designs, potentially compromising decision-making. Addressing this requires not only methodological innovation but also institutional collaboration. Embedding research within high-performance centers and promoting longitudinal case studies, particularly those that integrate real-world data from training and competition can help bridge this gap. These approaches maintain ecological validity while respecting the unique constraints of elite sport. Moreover, fostering a culture of data sharing among practitioners and researchers could amplify sample sizes across studies, supporting meta-analyses and enhancing generalizability. Ultimately, the pursuit of marginal gains in elite sport demands evidence that is both scientifically rigorous and practically relevant, a standard that can only be met through more collaborative and adaptive research methodologies.

### 4.2 Sex

Our findings reinforce the longstanding gender imbalance in sports science research (259), female athletes are significantly underrepresented in continuous modalities research involving RPE. This underrepresentation cannot be solely attributed to lower participation rates in sport, rather, it reflects entrenched methodological and cultural biases that have deprioritized the inclusion of women in high-performance research. A commonly cited barrier is the perceived complexity introduced by physiological, hormonal, and psychological fluctuations associated with the menstrual cycle, which can affect internal load markers such as RPE (260). This variability may lead some researchers to exclude female participants due to concerns over data consistency or increased analytical complexity (261). That has created a critical blind spot in our understanding of how women respond to training stimuli. However, this variability also underscores the importance of including women in RPE-focused research. Rather than being a limitation, menstrual cycle-related variability offers an opportunity to better understand internal load responses unique to female physiology.

However, our data, alongside emerging evidence (262), suggest that this variability is not a methodological problem but a key source of insight. For instance, the observed elevation in RPE during the luteal phase may reflect meaningful shifts in psychological and physiological load perception, which can inform more individualized training interventions. Rather than compromising study reliability, accounting for menstrual cycle phase through hormonal profiling or repeated measures can enhance the ecological validity and practical utility of RPE-based monitoring systems for female athletes. This has significant implications for coaching practice. RPE may serve as a low-cost, high-frequency tool to help athletes and practitioners modulate training intensity in relation to hormonal fluctuations, ultimately mitigating the risk of overtraining, injury, or burnout. Addressing this gender gap in monitoring research is not just a matter of representation, it is essential for developing evidence-based practices that reflect the diversity of athlete physiology (19).

To advance the field, researchers must prioritize studies that include female athletes, accounting for menstrual cycle phase through hormonal profiling, cycle tracking tools, or repeated measures. Incorporating these considerations would improve the ecological validity and applicability of RPE-based monitoring for female athletes.

While an exploration of the societal biases driving this issue falls outside the scope of our review, we strongly advocate for increased research efforts focused on female athletes. To address this gender gap, funders, researchers, and journal editors need to work together proactively and purposefully to make meaningful progress in this area.

### 4.3 Age

Our findings highlight an important limitation in the current application of RPE monitoring: its restricted age scope. While most studies focus on athletes aged 18–30, this reflects not only the concentration of elite performance within this range but also a systemic neglect of older adults who are increasingly active in competitive and recreational sport (263). This oversight has implications for both research validity and practical application. The physiological adaptations associated with aging, including diminished aerobic capacity, altered neuromuscular function, and hormonal changes fundamentally shift the perceptual experience of exertion (264). Consequently, applying RPE norms developed in younger populations to older adults risks misinterpreting training stress or underestimating perceived fatigue.

Moreover, existing psychometric validations of RPE scales often exclude master athletes, raising concerns about their construct validity in this cohort. It is possible that perceptual effort in aging populations is shaped not only by physiological inputs but also by psychosocial variables such as exercise self-efficacy, fear of injury, or training history. This aligns with emerging evidence suggesting that older individuals may report effort differently even at equivalent relative intensities (265). Therefore, we argue that future research must go beyond simply including older athletes—it must interrogate how aging modifies the perceptual constructs underlying RPE. This includes developing age-adjusted norms, validating scale responsiveness in this demographic, and integrating perceptual data into broader models of training load and recovery tailored to the master athlete.

Expanding the research focus in this way would not only improve the ecological validity of RPE for older adults but also facilitate safer and more effective training strategies across the lifespan. As sport becomes increasingly inclusive, it is essential that our monitoring tools evolve to meet the needs of all athletes—not just those at peak physiological performance.

### 4.4 Sports

Our findings underscore a significant imbalance in the application of RPE across continuous sports, with most studies focused on running, cycling, or swimming. While these sports lend themselves to internal load monitoring due to their accessibility, repetitive movement patterns, and physiological demands, the overrepresentation raises questions about the generalizability of RPE-based conclusions to other disciplines (262). Sports such as rowing, cross-country skiing, kayaking, and triathlons are each characterized by distinct biomechanical, technical, and environmental demands are underexplored, comprising only a fraction of the available literature. This gap is not merely academic; it carries practical implications for athletes and coaches operating in these contexts. The perceptual experience of exertion is inherently multimodal and context dependent. For example, upper-body dominant sports like kayaking or rowing may induce fatigue profiles that differ both physiologically and perceptually from those in predominantly lower-body sports (16). Similarly, sports involving complex coordination and environmental variability, such as cross-country skiing, may challenge the applicability of standard RPE protocols developed under more controlled conditions (266). If perceptual data are to inform training load, injury risk, or recovery management across disciplines, then sport-specific validation is essential to ensure both reliability and interpretability.

This oversight also reflects a methodological gap in the field: the implicit assumption that RPE operates uniformly across modalities. However, without direct evidence validating its use in diverse environments, this assumption may limit the tooĺs effectiveness. Future research should focus not only on expanding the range of sports studied but also on refining RPE methodologies to reflect the biomechanical, physiological, and cognitive demands of each discipline. Doing so will enhance the ecological validity of internal load monitoring and promote more inclusive, sport-specific training applications.

### 4.5 Factors influencing RPE

It is well-established that physiological and neural factors alone do not fully account for variability in RPE (9). A range of additional factors, including psychological, social, and environmental influences, also contribute to the variation in RPE (267). For instance, the presence of a co-actor and the nature of their involvement in the exercise, along with personality characteristics and current mood, have all been shown to impact RPE (268). Furthermore, individual factors such as age, gender, fitness level, and exercise experience can significantly modulate perceptions of exertion (269). Environmental factors also play a significant role in altering RPE, including external elements such as auditory stimuli, visual media feedback, and specific exercise instructions (270). Other influences include variations in RPE scales, the effects of hypnosis, ambient temperature, altitude, hydration status, and the intake of substances like caffeine, energy drinks, and even alcohol (271). For example, research has demonstrated that music can reduce perceived exertion during aerobic exercise by altering mood states (272), while other studies have highlighted the role of psychological factors like mood and stress in influencing RPE (71). Additionally, certain research has shown that factors such as the duration of high-intensity intervals, rather than the overall duration of a session, may have a stronger impact on RPE (270). Similarly, studies on the translation of RPE scales suggest that cross-cultural differences may affect how RPE is perceived, even when translation maintains internal consistency (273).

**Figure 3:**
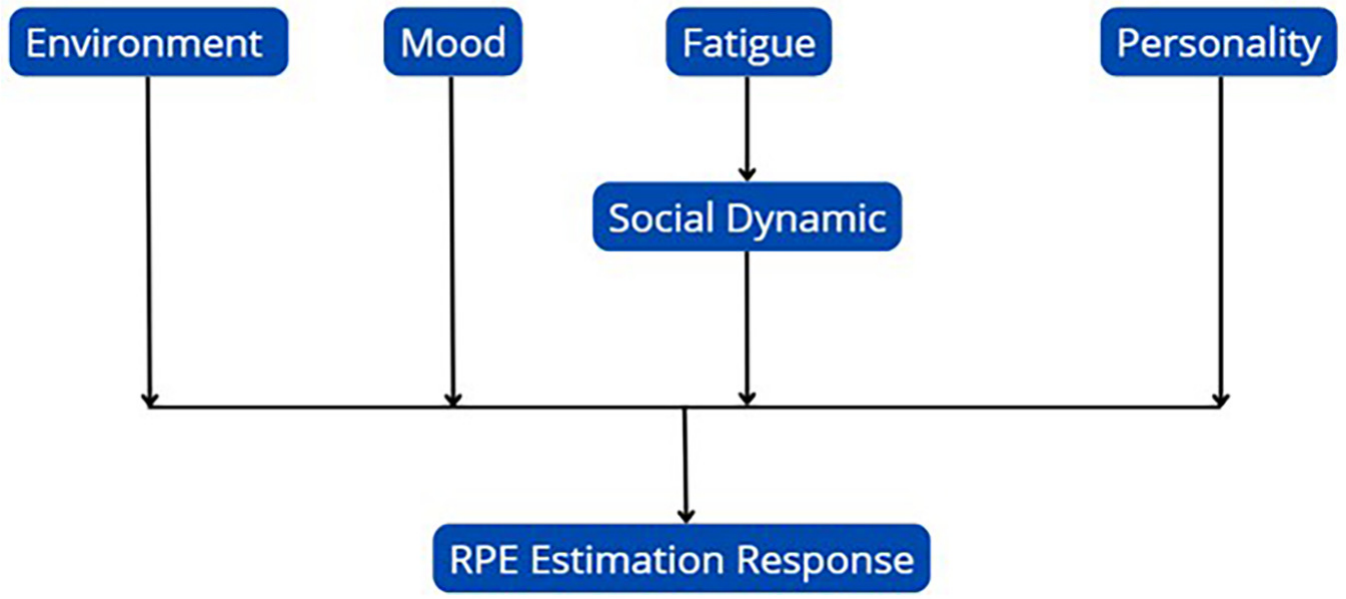
A conceptual model of internal and external moderators of RPE estimation responses.

Interestingly, research has indicated that factors like fatigue perception, stress, delayed onset muscle soreness (DOMS), and sleep do not substantially contribute to RPE during short-duration submaximal efforts or moderate training loads (274). This suggests that RPE is not entirely independent of afferent and efferent sensory signals, although such factors might play a more prominent role during higher-intensity or overtraining conditions, which require further investigation. Despite the influence of these numerous factors, a broad body of research affirms the validity of RPE as an effective indicator of exercise intensity. The strong reliability and internal consistency of RPE across a variety of sports, physical activities, and diverse populations spanning different age groups (children, adolescents, and adults), and varying levels of expertise, demonstrate its usefulness in monitoring training loads effectively. A conceptual model of internal and external moderators of RPE estimation responses is represented in Figure 3.

## 5 Future research

Despite RPE's broad application in numerous settings and contexts, some realms remain largely unexplored. This summary highlights opportunities for funders and researchers to focus under-researched areas of RPE, while potentially reducing research on topics that are already well-studied. On this basis, we accentuate the necessity of investigating sports other than those involving running, swimming, and cycling as the knowledge of their particular interactions with RPE is lacking. Furthermore, the development of sport-specific RPE protocols should be prioritized to improve to enhance specificity and accuracy of exertion assessments tailored to the unique demands of individual sports. Also, studies focused on elite athletes (tier 4 and 5) (255) are essential due to the unique characteristics of this population, as results from studies involving other types of athletes should not be extrapolated. While female athlete participation is increasing, their representation in research has not progressed at the same rate. It seems that RPE, especially due to its psychophysiological nature, can be significantly influenced by hormonal fluctuations that occur throughout the menstrual cycle. These hormonal changes, particularly in estrogen and progesterone levels, have been shown to affect both physical performance and subjective exertion, potentially leading to altered perceptions of effort during exercise (275). These changes highlight the need to use RPE as a valuable tool for monitoring internal load across menstrual cycle phases. Similarly, master athletes have been the subject of increasing interest over the years, but their representation in scientific literature remains limited. In fact, RPE can be greatly affected by age-related changes (276). Thus, it is critical to rigorously validate RPE in populations exhibiting greater daily variability in performance and subjective exertion assessment, such as female and master athletes, to ensure its reliability and applicability in these groups.

Additionally, the absence of longitudinal studies on RPE limits the ability to make meaningful intra-individual comparisons, which are currently more commonly used in practice. Longitudinal research would allow for a deeper understanding of how RPE changes over time within individuals, improving the accuracy and applicability of RPE assessments in monitoring training progress (277). To enhance the reproducibility and comparability of findings, researchers and practitioners should adopt a standardized reporting framework. This practice would facilitate the sharing of methodologies, data, and results, enabling more robust comparisons across studies and fostering collaborative advancements in the field. Without this, current comparisons may not capture full variability in RPE responses to long-term training adaptations or changes in fitness levels.

Finally, studies should be incentivized to disclose the characteristics of their subjects to add context for practitioners to be able to discuss and interpret their findings accurately.

## 6 Limitations

Although a comprehensive search was conducted in four databases, the exclusion of grey literature (e.g., theses, conference proceedings, or non-peer-reviewed studies) might have missed valuable insights. While scoping reviews do not typically assess methodological quality, the absence of this step means the findings rely on potentially low-quality evidence. Due to our preference for English-language publication, some studies published in other languages could potentially limit the scope of our findings and exclude alternative perspectives on RPE that would overall enrich our understanding of its application. By focusing exclusively on athletes of continuous modalities of exercise and establishing tier 2 as a minimum competitive level for inclusion, some well-trained athletes may have been left out. However, those were out of the scope of this review. Injured or unhealthy subjects were also excluded from the review therefore no comment can be made about the use of RPE in these settings. Additionally, including studies with small sample sizes may call into question the reliability of the findings due to concerns about statistical power, generalizability, and methodological rigor. Moreover, we recognize that heterogeneity in study design, population, and outcome measures, alongside variations in methodological quality (such as sample size and reporting rigor), may influence data interpretation. Finally, future studies should focus on reporting funding and competing interests to increase transparency with the scientific community.

## 7 Conclusions

In conclusion, this scoping review highlights the importance of RPE as a reliable tool for assessing exercise intensity across various populations and modes of exercise. However, the lack of longitudinal studies limits the understanding of how RPE changes over time, particularly in response to long-term training. RPE is extensively studied in some specific sports (e.g., cycling, swimming and running) while underrepresented in others (e.g., skiing and canoe Slalom). Additionally, we advocate for incentivize researchers to explore underrepresented populations in the literature (e.g., female; master and elite athletes). Finally, by requiring researchers to disclose their fundings status will promote transparency, accountability and trust in the scientific findings.

## Data Availability

The original contributions presented in the study are included in the article/Supplementary Material, further inquiries can be directed to the corresponding author.
