# Supplementary material for: Rating of perceived exertion in continuous sports: a scoping review with evidence gap map
Source: Front Sports Act Living. 2025 Jul 15;7:1553998. doi: 10.3389/fspor.2025.1553998 (PMC12303974; doi:10.3389/fspor.2025.1553998)
Supplement: Supplementary file 1 [file Datasheet1.docx]

***Supplementary Material***

# Supplementary Data

**Study Characteristics**

| **Author** | **Year** | **N** | **Gender** | **Competitive Tier** | **Main Outcome** |
| --- | --- | --- | --- | --- | --- |
| Abel et al. | 2019 | 8 | Male | Tier 3 | Physiological |
| Abellan-Aynés et al | 2019 | 10 | Unknown | Tier 2 | Physiological |
| Alfonso & Capdevilla | 2002 | 5 | Male | Tier 2 | Physiological |
| Al-Nawaiseh et al. | 2013 | 10 | Unknown | Tier 3 | Physiological |
| Alves et al. | 2023 | 13 | Male | Tier 2 | Performance |
| Astridge et al. | 2024 | 14 | Both | Tier 4 | Performance |
| Avila-Gandía et al. | 2021 | 5 | Male | Tier 4 | Performance |
| Avila-Gandía et al. | 2020 | 25 | Unknown | Tier 2 | Psychological |
| Azevedo et al | 2021 | 9 | Male | Tier 2 | Performance |
| Bahensky et al | 2020 | 16 | Both | Tier 2 | Performance |
| Bakhareva et al | 2022 | 20 | Unknown | Tier 3 | Training Monitoring |
| Baldassarre et al | 2024 | 8 | Both | Tier 4 | Training Monitoring |
| Balsalobre-Fernández et al. | 2014 | 15 | Both | Tier 3 | Training Monitoring |
| Balsalobre-Fernández et al. | 2015 | 15 | Both | Tier 3 | Training Monitoring |
| Barbosa et al. | 2020 | 20 | Both | Tier 3 | Performance |
| Barnes | 2017 | 25 | Male | Tier 3 | Training Monitoring |
| Barrero et al. | 2019 | 10 | Female | Tier 2 and 3 | Physiological |
| Barroso et al | 2014 | 160 | Both | Tier 2 and 3 | Training Monitoring |
| Barroso et al | 2015 | 13 | Unknown | Tier 2 | Training Monitoring |
| Barry et al | 2024 | 32 | Both | Tier 3,4 and 5 | Training Monitoring |
| Baur et al. | 2014 | 8 | Male | Tier 2 | Physiological |
| Beidaris & Platanou | 2017 | 18 | Both | Tier 2 | Performance |
| Bertochi et al. | 2025 | 52 | Both | Tier 2 | Training Monitoring |
| Bellinger et al. | 2020 | 14 | Both | Tier 3 | Training Monitoring |
| Best et al. | 2021 | 8 | Male | Tier 2 | Performance |
| Borg et al. | 2020 | 8 | Male | Tier 2 | Physiological |
| Borges et al. | 2020 | 18 | Male | Tier 2 | Training Monitoring |
| Borges et al. | 2014 | 10 | Both | Tier 3 | Training Monitoring |
| Born et al | 2020 | 21 | Both | Tier 3 | Performance |
| Bossi et al | 2023 | 13 | Male | Tier 2 | Physiological |
| Bossi et al | 2020 | 14 | Male | Tier 2 | Physiological |
| Boullosa et al | 2021 | 1 | Female | Tier 2 | Physiological |
| Brewer et al. | 2014 | 21 | Male | Tier 3 | Physiological |
| Brito et al. | 2017 | 9 | Male | Tier 4 | Physiological |
| Buechel et al | 2023 | 15 | Male | Tier 2 | Physiological |
| Casado et al. | 2019 | 16 | Both | Tier 3 and 4 | Performance |
| Cesanelli et al | 2021 | 8 | Male | Tier 2 | Physiological |
| Chamari et al. | 2016 | 11 | Male | Tier 4 | Psychological |
| Chauvineau et al | 2023 | 26 | Male | Tier 2 and 3 | Physiological |
| Chauvineau et al | 2024 | 26 | Male | Tier 2 | Physiological |
| Chiron et al. | 2024 | 1 | Female | Tier 5 | Physiological |
| Christen et al | 2016 | 15 | Both | Tier 2 | Training Monitoring |
| Clansey et al | 2012 | 21 | Male | Tier 2 | Biomechanical |
| Cochrane & Sleivert | 1999 | 8 | Male | Tier 2 | Physiological |
| Coelho et al | 2019 | 21 | Both | Tier 3 | Training Monitoring |
| Collette et al. | 2018 | 5 | Female | Tier 3 | Training Monitoring |
| Comotto et al. | 2015 | 16 | Both | Tier 3 | Psychological |
| Coquart et al. | 2012 | 23 | Male | Tier 2 | Psychological |
| Coquart et al. | 2009 | 27 | Male | Tier 2 and 3 | Physiological |
| Costa et al | 2012 | 6 | Male | Tier 2 | Physiological |
| Couto et al | 2015 | 19 | Male | Tier 2 | Performance |
| Craddock et al | 2020 | 119 | Both | Tier 2 | Training Monitoring |
| Crewe et al. | 2008 | 7 | Male | Tier 2 | Physiological |
| Cristina-Souza et al. | 2019 | 12 | Female | Tier 3 and 4 | Training Monitoring |
| Crowcroft et al | 2015 | 18 | Male | Tier 2 | Performance |
| Cruz et al. | 2019 | 75 | Both | Tier 2 | Performance |
| Cruz et al. | 2015 | 12 | Unknown | Tier 2 | Physiological |
| Cruz et al. | 2015 | 12 | Unknown | Tier 2 | Performance |
| Dai et al. | 2025 | 26 | Male | Tier 3 | Training Monitoring |
| D'Alleva et al | 2025 | 12 | Both | Tier 3 | Training Monitoring |
| D`Unienville et al | 2019 | 96 | Male | Tier 2 | Performance |
| Davies et al | 2018 | 8 | Both | Tier 2 | Training Monitoring |
| De Andrade Nogueira et al. | 2016 | 17 | Both | Tier 3 | Training Monitoring |
| De Andrade Nogueira et al. | 2015 | 17 | Both | Tier 2 | Training Monitoring |
| De lima Costa | 2020 | 12 | Male | Tier 3 and 4 | Physiological |
| De Moura et al. | 2021 | 14 | Both | Tier 2 | Physiological |
| DeBlauw et al | 2023 | 5 | Male | Tier 4 | Performance |
| Del Rosso et al. | 2021 | 27 | Male | Tier 3 | Performance |
| Delattre et al | 2006 | 7 | Unknown | Tier 2 and 3 | Physiological |
| DellaValle & Haas | 2012 | 48 | Female | Tier 3 and 4 | Performance |
| DellaValle & Haas | 2013 | 7 | Female | Tier 3 and 4 | Training Monitoring |
| Desgorces et al. | 2020 | 10 | Male | Tier 2 | Training Monitoring |
| Dijkhuis et al | 2020 | 23 | Both | Tier 2,3 and 4 | Training Monitoring |
| Dos Santos et al. | 2023 | 18 | Unknown | Tier 2 | Performance |
| Dotan et al. | 1989 | 15 | Male | Tier 2 | Physiological |
| Duc et al | 2020 | 14 | Male | Tier 2 | Performance |
| Easton et al | 2007 | 23 | Male | Tier 2 | Physiological |
| Edmonds et al | 2020 | 7 | Female | Tier 3 | Training Monitoring |
| Egan-Shutter et al | 2020 | 5 | Female | Tier 3 | Training Monitoring |
| Elmer et al. | 2010 | 18 | Male | Tier 2 | Performance |
| Faelli et al | 2019 | 22 | Male | Tier 2 | Training Monitoring |
| Ferreira-Junior et al | 2018 | 11 | Male | Tier 2 | Performance |
| Fidelis et al | 2024 | 8 | Both | Tier 3 | Physiological |
| Fleckenstein et al. | 2023 | 15 | Both | Tier 3 | Physiological |
| Flynn et al. | 1994 | 8 | Male | Tier 3 and 4 | Physiological |
| Fusco et al. | 2020 | 11 | Female | Tier 3 and 4 | Performance |
| Garatchea et al. | 2011 | 8 | Both | Tier 4 | Training Monitoring |
| Garcia et al | 2021 | 24 | Both | Tier 2 | Biomechanical |
| Garciá-Ramos et al. | 2015 | 17 | Both | Tier 4 | Training Monitoring |
| Garcin & Billat et al. | 2001 | 12 | Male | Tier 2 | Physiological |
| Garcin et al | 2006 | 25 | Both | Tier 2 | Training Monitoring |
| Garcin et al. | 2002 | 8 | Male | Tier 3 | Physiological |
| Greer et al | 2018 | 15 | Both | Tier 2 | Performance |
| Grego et al. | 2004 | 24 | Unknown | Tier 3 | Physiological |
| Gronwald | 2025 | 15 | Both | Tier 2 | Performance |
| Hadjicharalambous et al | 2006 | 18 | Male | Tier 2 | Performance |
| Hansen et al. | 2006 | 9 | Unknown | Tier 2 | Performance |
| Havemann et al. | 2006 | 8 | Male | Tier 3 | Physiological |
| Hernandéz-Cruz et al. | 2017 | 18 | Unknown | Tier 4 | Training Monitoring |
| Holgado et al. | 2019 | 28 | Male | Tier 2 | Psychological |
| Hottentorott et al. | 2022 | 24 | Both | Tier 2 | Performance |
| Huang et al | 2022 | 22 | Female | Tier 2,3 and 4 | Training Monitoring |
| Hursh et al | 2019 | 14 | Male | Tier 2 | Performance |
| Ieno et al. | 2021 | 4 | Both | Tier 4 | Training Monitoring |
| Inoue et al. | 2022 | 14 | Male | Tier 3 | Training Monitoring |
| Jasper et al. | 2024 | 9 | Both | Tier 3 | Performance |
| Jimenez-Reyes et al. | 2016 | 9 | Male | Tier 3 and 4 | Performance |
| Joseph et al. | 2008 | 20 | Both | Tier 2 | Performance |
| Kabasalakis et al. | 2020 | 21 | Both | Tier 2 | Physiological |
| Kaikkonen et al | 2012 | 13 | Male | Tier 2 | Physiological |
| Kaikkonen et al. | 2010 | 13 | Male | Tier 2 | Physiological |
| Kavouras et al | 2004 | 12 | Male | Tier 2 | Physiological |
| Kentta et al | 2014 | 11 | Both | Tier 3 | Training Monitoring |
| Kerherve et al | 2015 | 15 | Male | Tier 2 | Performance |
| Kesisoglou et al. | 2021 | 11 | Both | Tier 2 | Physiological |
| Kirwan et al | 1998 | 10 | Male | Tier 2 | Performance |
| Kjosen Taslnes et al. | 2024 | 14 | Male | Tier 3 | Physiological |
| Kruschewsky et al. | 2018 | 9 | Male | Tier 2 | Performance |
| Laden et al | 2019 | 8 | Male | Tier2 | Performance |
| Lamberts | 2014 | 102 | Both | Tier 2,3 and 4 | Performance |
| Lamberts et al | 2009 | 1 | Male | Tier 4 | Performance |
| Latorre-Roman et al. | 2022 | 33 | Male | Tier 2 | Training Monitoring |
| Le Douairon Lahaye et al. | 2022 | 7 | Female | Tier 3 | Physiological |
| Learsi et al | 2019 | 9 | Male | Tier 2 | Performance |
| Li et al | 2020 | 19 | Both | Tier 2 | Biomechanical |
| Lucia et al | 2004 | 8 | Male | Tier 3 and 4 | Performance |
| Maclejewski et al. | 2007 | 10 | Male | Tier 4 | Physiological |
| Manchado-Gobatto et al. | 2014 | 6 | Unknown | Tier 3 and 4 | Physiological |
| Mann et al. | 2019 | 15 | Female | Tier 2 | Training Monitoring |
| Mann et al. | 2015 | 10 | Both | Tier 2 | Physiological |
| Manzi et al. | 2015 | 7 | Male | Tier 2 | Training Monitoring |
| Mateo-March et al. | 2022 | 102 | Unknown | Tier 4 | Training Monitoring |
| Mateus et al. | 2022 | 20 | Male | Tier 2 | Biomechanical |
| Matos et.al | 2020 | 25 | Male | Tier 3 | Training Monitoring |
| Matos et.al | 2021 | 25 | Male | Tier 3 | Training Monitoring |
| Matos et.al | 2019 | 47 | Male | Tier 3 | Training Monitoring |
| Mcgawley et al | 2017 | 20 | Both | Tier 3 | Training Monitoring |
| Mickleborough et al | 2009 | 24 | Both | Tier 2 | Physiological |
| Molinari et al. | 2020 | 12 | Both | Tier 2 | Training Monitoring |
| Munoz & Varela-Sanchez | 2018 | 1 | Male | Tier 2 | Training Monitoring |
| Nagle et al. | 2015 | 19 | Female | Tier 4 | Physiological |
| Nakamura et al | 2009 | 8 | Male | Tier 3 and 4 | Training Monitoring |
| Napier et al | 2020 | 68 | Both | Tier 2 | Training Monitoring |
| Neto et al | 2021 | 11 | Both | Tier 2 | Training Monitoring |
| Nicolas et al. | 2019 | 83 | Both | Tier 3 | Physiological |
| Nicolò et al. | 2016 | 9 | Male | Tier 3 | Physiological |
| Nikitakis et al. | 2025 | 12 | Male | Tier 3 | Training Monitoring |
| Nikolopoulos et al | 2004 | 8 | Male | Tier 2 | Physiological |
| Nugent et al. | 2018 | 10 | Both | Tier 3 | Training Monitoring |
| O´Connor et al. | 1991 | 40 | Both | Tier 3 | Physiological |
| Olivier & Scott | 1993 | 11 | Male | Tier 2 | Physiological |
| Otter et al | 2022 | 23 | Both | Tier 3 and 4 | Training Monitoring |
| Palmer et al | 1999 | 6 | Male | Tier 2 | Performance |
| Parry et al | 2012 | 15 | Unknown | Tier 2 | Performance |
| Parry et al. | 2011 | 12 | Both | Tier 3 | Psychological |
| Peinado et al | 2018 | 14 | Male | Tier 2 and 3 | Performance |
| Pérez-Landaluce et al. | 2002 | 72 | Unknown | Tier 2 and 3 | Physiological |
| Peserico et al. | 2015 | 20 | Male | Tier 2 | Physiological |
| Piatrikova et al | 2021 | 10 | Both | Tier 3 | Training Monitoring |
| Piero et al | 2018 | 9 | Both | Tier 2 | Physiological |
| Pind et al. | 2021 | 19 | Both | Tier 4 | Physiological |
| Pind et al. | 2023 | 13 | Both | Tier 3 | Training Monitoring |
| Pind et al. | 2021 | 19 | Both | Tier 3 | Training Monitoring |
| Pinot & Grappe | 2015 | 1 | Male | Tier 4 | Training Monitoring |
| Pirscoveanu & Oliveira | 2023 | 43 | Both | Tier 2 | Training Monitoring |
| Pollastri et al | 2021 | 8 | Male | Tier 4 | Performance |
| Pope et al | 2023 | 16 | Both | Tier 2 | Performance |
| Portillo & Rodriguez | 2020 | 13 | Both | Tier 3 | Physiological |
| Potteiger & Weber | 1994 | 9 | Male | Tier 3 | Physiological |
| Quittman et al. | 2020 | 12 | Male | Tier 3 | Biomechanical |
| Rietjens et al. | 2005 | 7 | Male | Tier 2 | Training Monitoring |
| Riska et al | 2024 | 23 | Unknown | Tier 4 | Physiological |
| Roberts et al. | 2022 | 9 | Male | Tier 2 | Physiological |
| Rodríguez et al. | 2015 | 54 | Both | Tier 4 | Performance |
| Rodriguez-Marroyo et al. | 2023 | 25 | Both | Tier 3 | Physiological |
| Rodriguez-Marroyo et al. | 2013 | 12 | Unknown | Tier 3 | Training Monitoring |
| Rodriguez-Marroyo et al. | 2012 | 12 | Unknown | Tier 3 | Training Monitoring |
| Rodriguez-Medina et al. | 2025 | 66 | Male | Tier 3 | Physiological |
| Rojas-Valverde et al. | 2023 | 23 | Both | Tier 3 | Physiological |
| Roos et al | 2018 | 85 | Both | Tier 2 and 3 | Training Monitoring |
| Ryan et al | 2020 | 12 | Male | Tier 2 | Training Monitoring |
| Sanchez-Otero et al. | 2022 | 11 | Male | Tier 3 | Performance |
| Sanders et al. | 2018 | 12 | Male | Tier 4 | Training Monitoring |
| Sanders et al. | 2017 | 15 | Both | Tier 4 | Training Monitoring |
| Sanders et al. | 2019 | 30 | Both | Tier 4 | Performance |
| Schmitz | 2020 | 12 | Both | Tier 2 | Physiological |
| Schoenmakers & Reed | 2019 | 12 | Male | Tier 2 | Physiological |
| Seiler & Sjursen | 2004 | 12 | Both | Tier 2 | Physiological |
| Seiler & Sylta | 2017 | 63 | Male | Tier 3 | Physiological |
| Shannon et al | 2016 | 8 | Male | Tier 2 | Performance |
| Sharma et al. | 2018 | 8 | Both | Tier 3 | Performance |
| Sharma et al. | 2017 | 19 | Both | Tier 3 | Performance |
| Shaver et al | 2018 | 19 | Female | Tier 2 | Performance |
| Sherman et al. | 2022 | 36 | Female | Tier 4 | Training Monitoring |
| Silva et al | 2014 | 11 | Male | Tier 3 | Performance |
| Sixsmith et al | 2023 | 10 | Both | Tier 3 and 4 | Training Monitoring |
| Smith et al. | 2023 | 14 | Both | Tier 3 | Physiological |
| Soriano et al | 2021 | 19 | Male | Tier 3 | Physiological |
| Stellingwerf | 2012 | 3 | Male | Tier 3 | Training Monitoring |
| Stevens et al | 2017 | 7 | Male | Tier 2 | Physiological |
| Surala et al | 2023 | 18 | Both | Tier 3 and 4 | Training Monitoring |
| Swart et al. | 2012 | 7 | Male | Tier 3 | Physiological |
| Sylva & Byrd | 1990 | 17 | Both | Tier 3 | Physiological |
| Talsnes et al. | 2020 | 24 | Both | Tier 3 | Training Monitoring |
| Terrazas et al | 2019 | 10 | Male | Tier 2 | Physiological |
| Thomas et al. | 2012 | 10 | Male | Tier 2 | Performance |
| Tomar & Allen | 2019 | 12 | Male | Tier 3 | Training Monitoring |
| Toubekis et al. | 2013 | 12 | Both | Tier 3 | Performance |
| Tran et al. | 2015 | 14 | Both | Tier 3 | Training Monitoring |
| Tumilty et al. | 2014 | 7 | Male | Tier 3 | Physiological |
| Turner and Rice | 2021 | 10 | Male | Tier 3 | Performance |
| Vacher et al | 2017 | 16 | Both | Tier 3 | Psychological |
| Van Erp & Sanders | 2021 | 20 | Male | Tier 4 | Training Monitoring |
| Van erp et al | 2022 | 15 | Female | Tier 4 | Training Monitoring |
| Van Erp et al. | 2019 | 21 | Male | Tier 4 | Training Monitoring |
| Van Erp et al. | 2019 | 21 | Male | Tier 4 | Training Monitoring |
| Vaz et al. | 2014 | 8 | Male | Tier 2 | Physiological |
| Viana et al. | 2016 | 19 | Male | Tier 3 | Training Monitoring |
| Viribay et al. | 2020 | 20 | Male | Tier 3 and 4 | Physiological |
| Vitale et al. | 2022 | 5 | Both | Tier 5 | Training Monitoring |
| Voet et al. | 2022 | 11 | Both | Tier 2 and 3 | Training Monitoring |
| Voet et al | 2025 | 10 | Male | Tier 3 | Training Monitoring |
| Wahl et al. | 2021 | 6 | Male | Tier 3 | Physiological |
| Wallace et al. | 2014 | 7 | Unknown | Tier 3 | Training Monitoring |
| Wallace et al. | 2009 | 12 | Both | Tier 3 | Training Monitoring |
| Warr-di Piero et al.. | 2018 | 19 | Both | Tier 2 | Performance |
| Wilson et al | 2013 | 15 | Both | tier 4 | Performance |
| Wittig et al. | 1989 | 10 | Male | Tier 2 | Training Monitoring |
| Yang et al. | 2024 | 10 | Both | Tier 4 | Training Monitoring |
| Yogev et al. | 2023 | 21 | Both | Tier 3 | Physiological |
| Yoma et al. | 2021 | 31 | Both | Tier 3 | Training Monitoring |
| Yoma et al. | 2021 | 16 | Both | tier 2 and 3 | Training Monitoring |
| Yu et al | 2023 | 8 | Male | Tier 4 | Training Monitoring |
| Zagatto et al. | 2016 | 11 | Male | Tier 2 | Physiological |


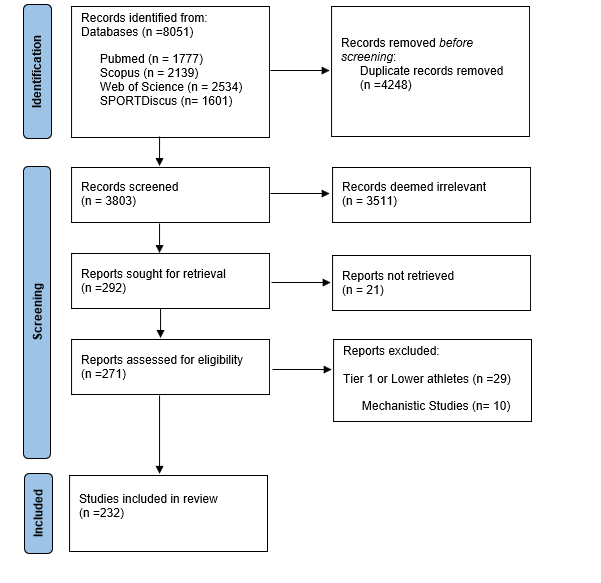


Figure 1 – PRISMA flow diagram used for the article search.

Table 1- Representation of RPE application method, key outcome, measures and average sample size by Sport.

| Sport | RPE application method | Key Outcome | Measures | Average Sample Sizes |
| --- | --- | --- | --- | --- |
| Running | RPE Estimation | Physiological | RPE, HR, Bla, VO2 | 17 |
| Cycling | RPE Estimation | Performance | RPE, HR, Bla, Power Output | 23 |
| Swimming | RPE Estimation | Training Monitoring | RPE, HR, Bla, time trial | 19 |
| Rowing | Session RPE Method | Training Monitoring | RPE, HR, Bla | 17 |
| Triathlon | RPE Estimation | Physiological | RPE, HR, Bla | 15 |
| Skiing | RPE Estimation | Performance | RPE, HR, Bla | 12 |
| Kayak | RPE Estimation | Physiological | RPE, HR, BLa | 9 |
| Canoe Slalom | Session RPE Method | Training Monitoring | RPE, HRV | 21 |


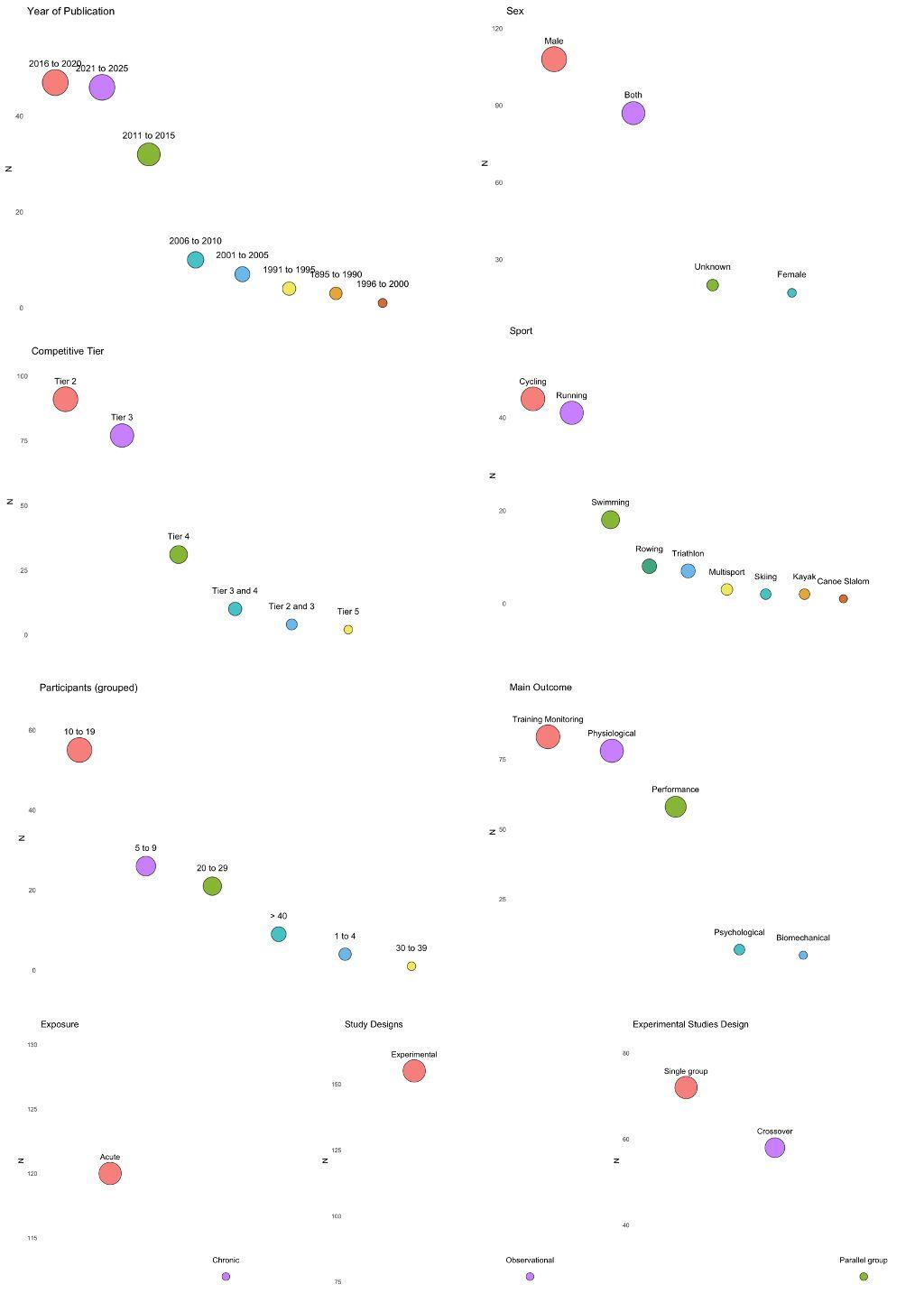


Figure 2- EGM of RPE research in continuous modes of exercise in healthy athletes. Rows represent primary outcome measures. Circle size within each cell reflects the relative magnitude of evidence for a given outcome compared to others, with larger circles indicating greater study volume and smaller circles fewer studies.


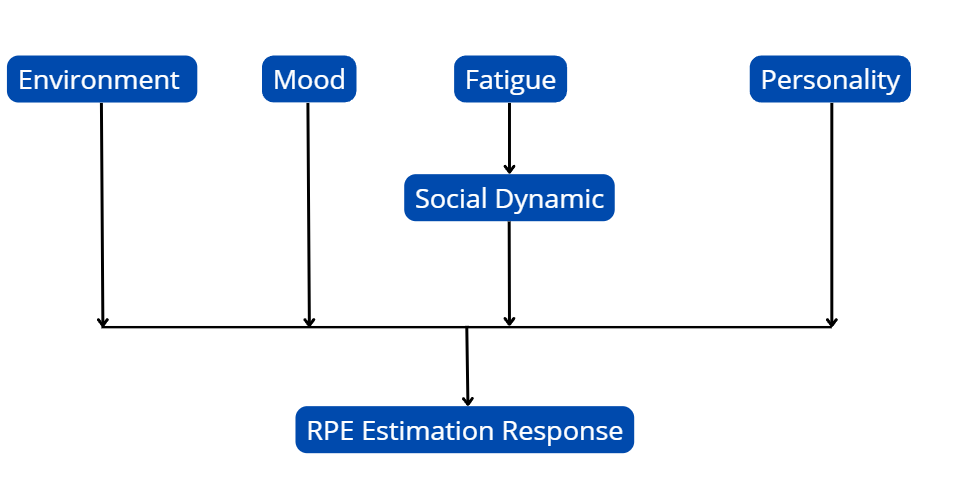
Figure 3- Conceptual model of Internal and external moderator of RPE estimation responses
